# Hepatoid carcinoma of the ovary: a literature review

**DOI:** 10.3389/fonc.2026.1970223

**Published:** 2026-09-21

**Authors:** Mingyuan Yuan, Jieqiong Jin, Chengjuan Jin, Xiaoyan Bai

**Affiliations:** 1 Department of Gynecology, Jiaozhou Central Hospital of Qingdao, Qingdao, China; 2 Department of Obstetrics and Gynecology, Shanghai General Hospital, School of Medicine, Shanghai Jiao Tong University, Shanghai, China

**Keywords:** alpha-fetoprotein, hepatoid carcinoma, immunohistochemistry, ovary, pathological features

## Abstract

Hepatoid carcinoma of the ovary (HCO) exhibits morphological and immunophenotypic features highly similar to those of hepatocellular carcinoma. Immunohistochemical staining for alpha-fetoprotein, hepatocyte paraffin antigen 1, and glypican-3 is an important auxiliary approach for the clinical diagnosis of HCO. Currently, most treatment regimens for this disease are developed with reference to clinical management protocols for high-grade epithelial ovarian cancer. Hepatoid carcinoma of the ovary (HCO) is an extremely rare, distinct histological subtype of primary ovarian adenocarcinoma that exhibits remarkable clinicopathological similarity to hepatocellular carcinoma (HCC). The histogenetic origin of HCO remains controversial, with hypotheses proposing derivation from ovarian surface epithelium or extragonadal non-germ cell precursors. This neoplasm primarily affects postmenopausal women, typically presenting as unilateral or bilateral cystic-solid ovarian masses accompanied by significantly elevated serum alpha-fetoprotein (AFP) levels. HCO is characterized by high biological aggressiveness, with most cases diagnosed at an advanced stage leading to a uniformly poor prognosis. Owing to its extreme rarity, existing research on HCO is largely limited to sporadic case reports. This systematic review aggregates eligible HCO cases published from 1987 to January 2026, conducts pooled analysis and comprehensive synthesis of clinical characteristics, pathological features, treatment strategies and molecular findings, with the aim of providing evidence-based references for clinical diagnosis and therapeutic decision-making, and establishing a theoretical foundation for future translational research.

## 1 Introduction

Hepatoid adenocarcinoma (HAC) is a rare extrahepatic adenocarcinoma characterized by prominent hepatocellular differentiation. To date, HAC has been reported across multiple anatomical sites, including the gastrointestinal tract, ovary, pancreas, lung, kidney, uterus, and urinary bladder. Histologically and immunophenotypically, HAC closely resembles hepatocellular carcinoma (HCC) but differs substantially from conventional adenocarcinomas. First described by Ishikura et al. in 1987, hepatoid carcinoma of the ovary (HCO) is an extremely rare and highly lethal subtype of ovarian neoplasms. Owing to its exceedingly low incidence, HCO has not been included in the current standardized classification system for ovarian tumors. Its rarity combined with distinct pathological features presents considerable challenges to clinical diagnosis and therapeutic management. A systematic literature search was performed to identify eligible studies published up to January 2026. After excluding review articles and duplicate case reports, a total of 41 qualifying publications enrolling 48 patients with HCO were included in the analysis. Drawing on aggregated clinical data, this review systematically summarizes and analyzes the histogenetic origin, clinical manifestations, histopathological and immunohistochemical features, differential diagnosis, therapeutic strategies, and prognostic determinants of HCO, with the aim of improving the understanding of this rare ovarian malignancy among clinicians and pathologists.

## 2 Oncogenesis of hepatoid carcinoma of the ovary

### 2.1 Histogenetic origin

The histological origin of hepatoid carcinoma of the ovary (HCO) remains a subject of ongoing debate. Ishikura, Scully et al. proposed that HCO originates from hepatocellular metaplasia of the ovarian surface epithelium (1). The epidemiological characteristics of HCO are consistent with those of epithelial ovarian carcinoma: the disease primarily affects postmenopausal women, and no association with ovarian dysgenesis has been identified. In a subset of HCO cases, the tumor harbors mixed components of serous, mucinous, or endometrioid carcinoma (2–6), which further supports the hypothesis of an ovarian surface epithelial origin. Most patients with HCO present with elevated serum CA125 levels, a finding corroborated by the immunohistochemical detection of CA125 overexpression in tumor tissues.

The cytokeratin expression profile of HCO further supports an epithelial origin: HCO exhibits an immunophenotype of CK18+/CK19+/CK20±, which differs from the canonical hepatocyte signature (CK18+/CK19-/CK20-) but aligns with that of conventional ovarian epithelial adenocarcinomas (7). Collectively, most scholars hold that HCO derives from ovarian surface epithelial cells with multipotent differentiation capacity, which acquire a hepatoid phenotype via metaplasia or transdifferentiation. An alternative hypothesis suggests that HCO arises from hepatoid differentiation of germ cell origin (6). Notably, D’Antonio reported a case of HCO concurrent with a sex cord-stromal tumor, indicating a possible mixed histogenetic origin (4). In addition, multiple reported HCO cases have been associated with pregnancy, chronic hepatitis, or pancytopenia, suggesting that HOC has multifactorial pathogenesis (4, 8–11).

### 2.2 Molecular mechanisms

Due to the low incidence of hepatoid adenocarcinoma (HAC), molecular profiling studies on this disease remain scarce, with investigations on gastric hepatoid adenocarcinoma being the most abundant among existing literature. Cumulative evidence has confirmed that the tumor suppressor gene TP53 plays a core role in the phenotypic transformation of hepatoid adenocarcinoma of the stomach (HAS), and TP53 has the highest mutation frequency across all HAC subtypes (12–14). It is also recognized as one of the key drivers of the tumorigenesis and malignant biological behavior of hepatoid carcinoma of the ovary (HCO) (6) (Zhang et al., 2025). In contrast, other common driver mutations such as BRCA1/2 are typically negative in HCO (6, 11, 15).

Among the rare cases with comprehensive molecular profiling performed, several potentially pathogenic germline mutations have been identified. For example, Zhang et al. (15) conducted whole-exome sequencing (WES) on a patient who achieved a favorable response to a PARP inhibitor combined with an antiangiogenic agent, and identified a novel frameshift mutation in the BLM gene (NM_000057.2, c.1290_1291delinsatcaggcctccatag, p.Y430fs1), which can induce homologous recombination repair deficiency (HRD). In addition, somatic mutations including PIK3C3 (c.2183G>C, p.S728T) and the *TERT* promoter region (c.-124C>T) have also been detected in HCO tumor tissues. These findings indicate that abnormal activation of the PI3K-AKT pathway and aberrant telomerase activity may contribute to the pathogenesis of HCO (11).

Existing molecular evidence provides clues for clarifying the histological origin of HCO. Although HCO mimics hepatocellular carcinoma (HCC) morphologically, its molecular profile is more similar to that of epithelial ovarian carcinoma (EOC). WES data from Zhang et al. (15) showed that the somatic mutation spectrum of the patient overlapped with the mutation profile of ovarian adenocarcinoma in the COSMIC database (e.g., SMAD4), while no overlap was observed with the mutation spectrum of hepatocellular carcinoma. This finding supports the hypothesis at the molecular level that HCO may originate from the ovarian surface epithelium, and the hepatoid phenotype is acquired via transdifferentiation or metaplasia. Furthermore, similar to most high-grade serous ovarian cancers, HCO is often characterized by microsatellite stability (MSS) and low tumor mutation burden (TMB) (11, 15). In addition, the focal PAX8 expression observed in HCO supports the origin of this tumor from the Müllerian duct (13). Large-scale genomic cohort studies are urgently needed to characterize the full molecular profile of HCO, which will provide novel insights into HCO histogenesis and establish a molecular foundation for precise targeted therapy.

## 3 Clinical characteristics of HCO

HCO predominantly affects middle-aged and elderly women, with a reported age range of 27–78 years and a median age of 55.5 years. Perimenopausal and postmenopausal women account for approximately 80% of all cases, which is consistent with the epidemiological profile of conventional epithelial ovarian carcinoma. To date, only one case of gestational HCO has been documented: a 35-year-old patient presented with an abdominal mass at 23 weeks of gestation and received a postoperative diagnosis of HCO (8). Owing to the limited number of reported cases and the sporadic nature of HCO occurrence, its exact incidence, geographical variation, and ethnic predilection remain unclear.

The clinical manifestations of hepatoid carcinoma of the ovary (HCO) are non-specific and frequently mimic those of conventional epithelial ovarian tumors. The primary clinical symptoms include abdominal pain (58.33%, 28/48), abdominal distension (45.83%, 22/48), palpable abdominal mass (31.25%, 15/48), weight loss (12.5%, 6/48), and vaginal bleeding (8.33%, 4/48). Patients with advanced HCO frequently develop metastatic complications, which can present with gastrointestinal symptoms (anorexia, nausea, vomiting, hematochezia) and thoracic metastatic manifestations (dyspnea) (2, 10, 16–22). Physical examination typically detects pelvic or lower abdominal masses, and approximately one-third of patients present with massive ascites characterized by shifting dullness on percussion.

Imaging modalities for the evaluation of HCO include ultrasonography, computed tomography (CT), magnetic resonance imaging (MRI) and positron emission tomography-computed tomography (PET-CT). The solid component of HCO demonstrates attenuation comparable to that of normal liver parenchyma, with either clear or ill-defined boundaries, hypervascularity in partial lesions, and cystic areas corresponding to necrosis and hemorrhage. Contrast-enhanced CT and MRI reveal moderate enhancement of solid components and no enhancement of cystic necrotic areas, and concomitant intraperitoneal metastasis is observed in some cases. PET-CT demonstrates elevated standardized uptake value (SUV) and hypermetabolic activity in HCO lesions. Preoperative imaging findings of HCO lack specific distinguishing features, rendering HCO prone to misdiagnosis as conventional epithelial ovarian carcinoma. Nevertheless, imaging modalities can aid in the exclusion of primary hepatic malignancies.

Elevated serum alpha-fetoprotein (AFP) is a relatively specific laboratory hallmark of hepatoid carcinoma of the ovary (HCO). Among 40 patients with available AFP data, 36 (90%) presented with marked AFP elevation, ranging from 74 ng/mL to 329,732 ng/mL with a median value of 2,475 ng/mL; 12 patients (30%) had AFP levels exceeding 20,000 ng/mL, with one extreme case reaching over 300,000 ng/mL (16). Serum AFP levels decline markedly after surgical resection, and rise again before tumor recurrence, confirming its diagnostic value as well as utility as a surveillance biomarker for recurrence. Notably, 4 cases (10%) presented with normal AFP levels, and diagnosis relied exclusively on morphological and immunohistochemical findings (17, 22–24). Clinicians should maintain vigilance for AFP-negative occult HCO.

Approximately 91.89% of HCO patients exhibited elevated serum Cancer Antigen 125 (CA125) (34/37, range 37.5–20,164 U/mL). Serum Cancer Antigen 19-9 (CA19-9), Cancer Antigen 153 (CA153) and carcinoembryonic antigen (CEA) levels were normal or mildly elevated in most cases. One case of AFP-negative HCO showed marked elevation of both CA153 (735.52 U/mL) and CA19-9 (162.80 U/mL) (15), while a 36-year-old patient presented with significantly increased CA19-9 (835 U/mL) (25). Human epididymis protein 4 (HE4), a well-validated biomarker for epithelial ovarian carcinoma, was only found to be elevated in one HCO case, which also presented with concurrent CA125 elevation (26). Serum beta-human chorionic gonadotropin (β-HCG) is rarely abnormal in HCO, with only one tested case showing mild elevation (125 U/mL) (27). Rarely reported tumor markers including inhibitor of apoptosis protein (IAP), tissue polypeptide antigen (TPA) and lactate dehydrogenase (LDH) have also been reported to be abnormally elevated in individual HCO cases (7).

## 4 Pathological features and immunohistochemistry

Among the 44 cases with complete pathological records, unilateral tumor involvement accounted for 68.18% (30/44), while bilateral involvement accounted for 31.81% (14/44). Tumor diameter ranged from 5 cm to 35 cm, with a median diameter of 10 cm. Of note, one case presented with normal bilateral ovarian volume, which warrants clinical vigilance (22). Macroscopically, most tumors were exophytic, irregular with nodular contours, and presented a solid or cystic-solid architecture. On cut section, solid areas showed a whitish to yellowish appearance, while cystic cavities contained hemorrhagic brown fluid.

Microscopically, tumor cells were arranged in sheet-like, trabecular, nested or cord-like patterns, with abundant sinusoidal vasculature; focal glandular or bile duct-like structures, hyaline degeneration, hemorrhage, and necrosis were frequently observed. Tumor cells exhibited moderate-to-severe atypia, with occasional bizarre giant cells, abundant eosinophilic or clear cytoplasm, polygonal to round nuclei, prominent nucleoli, and frequent mitotic figures. Approximately 41.30% of cases contained amylase-resistant, PAS-positive eosinophilic hyaline globules. Focal yellow-green pigment deposition was identified in a subset of cases, which was confirmed as bile via Hall stain (1, 9, 23, 27, 28). In 10.41% of cases, hepatoid carcinoma of the ovain (HCO) was admixed with other histological components, including serous carcinoma, mucinous carcinoma, endometrioid carcinoma, sex cord-stromal tumor, and clear cell carcinoma (2–6).

Hepatoid carcinoma of the ovary (HCO) exhibits a hybrid immunophenotype with co-expression of both hepatocellular and ovarian epithelial biomarkers. Alpha-fetoprotein (AFP), the canonical specific marker of hepatocellular carcinoma (HCC), tested positive in over 90% of reported HCO cases and serves as a core diagnostic indicator, with only 8.33% of cases exhibiting AFP negativity (17, 22, 24, 29). The expression rates of other common hepatocyte differentiation markers in HCO are as follows: Glypican-3 (GPC-3, 76.92%), HepPar-1 (78.26%), alpha-antitrypsin (ACT, 100%), albumin (ALB, 100%), alpha 1-antitrypsin (α1-AT, 92.3%), and Arg1 (75%), which are consistent with previously published literature (30) (note: duplicate entry of HepPar-1 is retained as in the original text).

HCO partially expresses epithelial tumor markers: epithelial membrane antigen (EMA, 76.92%), cancer antigen 125 (CA125, 54.54%) and carcinoembryonic antigen (CEA, 68.42%). Most germ cell tumor markers are negative in HCO, while placental alkaline phosphatase (PLAP) has a 50% positive rate. Markers including OCT3/4(OCT4), CD117, human placental lactogen (HPL) and human chorionic gonadotropin (HCG) are largely negative, with only one reported HCO case testing positive for HCG (1). Spalt-like transcription factor 4 (SALL4) is a well-established critical immunohistochemical biomarker for germ cell tumors, and its role has been extensively investigated in hepatoid adenocarcinoma (HAC) of the gastrointestinal tract.

Current evidence consistently demonstrates that SALL4 expression is absent in hepatocellular carcinoma but strongly positive in hepatoid adenocarcinoma (14). Notably, published data show that SALL4 achieves a sensitivity of 87% and a specificity of 100% for distinguishing hepatoid adenocarcinoma from hepatocellular carcinoma, making it a highly reliable diagnostic adjunct to differentiate primary or metastatic hepatoid adenocarcinoma from metastatic hepatocellular carcinoma (14). However, unlike its well-characterized status in HCC, current evidence regarding SALL4 expression in HCO remains limited, with available studies reporting a 55.55% positive expression rate. Additional well-designed investigations are therefore warranted to clarify its diagnostic utility and biological relevance in HCO.

Most sex cord-stromal tumor markers are negative in HCO: Inhibin has a 33.3% (3/9) positive rate, WT1 has a 16.66% (1/6) positive rate, and Calretinin (CR) is universally negative. Cytokeratins are highly expressed in HCO: pan-cytokeratin (pan-CK) has an 80% (8/10) positive rate, AE1/AE3 has a 75% positive rate, and only one reported case tested positive for CAM5.2 (31). The positivity rates of cytokeratin subtypes are as follows: CK8 (100%), CK18 (90%), CK7 (57.89%), CK19 (28.57%) and CK20 (14.28%). Positive expression of CK19 and CK20 was exclusively detected in HCO cases with admixed serous, endometrioid or mucinous carcinoma components (3). Proliferation and tumor suppressor markers including Ki67, P53 (88.88%) and P16 (40%) showed variable expression across cases. Biomarkers including estrogen receptor (ER), progesterone receptor (PR), paired box 8 (PAX8), D2-40, S-100, synaptophysin (Syn), Napsin A and thyroid transcription factor 1 (TTF-1) were consistently negative in all reported HCO cases. CDX2, a diagnostic marker for gastric hepatoid adenocarcinoma, is not expressed in HCO, indicating that hepatoid adenocarcinoma across different organs has heterogeneous pathogenetic mechanisms (13, 17). CD10 tested positive in 60% of HCO cases, suggesting primitive tubular differentiation, while CD30 was universally negative.A summary of common immunohistochemical findings for HCO is presented in **Table 1**.

**Table 1:** Common immunohistochemical expression profiles of HCO.

Case	Pub.Year	Author	AFP	HepPar-1	Glypican-3	SALL4	Arg1	AAT (α1-AT)	ACT	ALB	CA125	CEA	EMA	CK7	CK8	CK18	CK19	CK20	AE1/AE3	PLAP	CD10
1 (1)	1987	Ishikuraet al.	+	ND	+	ND	ND	+	+	+	-	+	ND	ND	ND	ND	ND	ND	ND	ND	ND
2			+	ND	-	ND	ND	+	+	+	+	-	ND	ND	ND	ND	ND	ND	ND	ND	ND
3			+	ND	-	ND	ND	+	+	+	-	+	ND	ND	ND	ND	ND	ND	ND	ND	ND
4			+	ND	-	ND	ND	+	+	+	-	-	ND	ND	ND	ND	ND	ND	ND	ND	ND
5			+	ND	+	ND	ND	-	+	+	+	+	ND	ND	ND	ND	ND	ND	ND	ND	ND
6 (7)	1991	Matsutaet al.	+	ND	ND	ND	ND	ND	ND	ND	ND	ND	ND	ND	ND	ND	ND	ND	ND	ND	ND
7 (32)	1993	Tamakoshiet al.	+	ND	ND	ND	ND	ND	ND	ND	ND	ND	ND	ND	ND	ND	ND	ND	ND	ND	ND
8 (33)	1993	Badreddine Jet al.	+	ND	ND	ND	ND	ND	ND	ND	ND	ND	ND	ND	ND	ND	ND	ND	ND	ND	ND
9 (34)	1995	Nishidaet al.	+	ND	ND	ND	ND	ND	ND	+	ND	+	+	ND	ND	ND	ND	ND	ND	ND	ND
10 (2)	1996	Scurryet al.	+	ND	ND	ND	ND	ND	ND	ND	ND	ND	ND	ND	ND	ND	ND	ND	ND	ND	ND
11 (35)	1998	Trivediet al.	+	ND	ND	ND	ND	+	ND	ND	ND	+	+	ND	ND	ND	ND	ND	ND	ND	ND
12 (8)	1998	Maymonet al.	+	ND	ND	ND	ND	ND	ND	ND	ND	ND	ND	ND	ND	ND	ND	ND	ND	ND	ND
13 (9)	1999	Senzakiet al.	+	ND	ND	ND	ND	+	+	ND	ND	+	ND	+	+	+	ND	ND	ND	ND	ND
15 (36)	2002	Leeet al.	+	ND	ND	ND	ND	ND	ND	ND	ND	ND	ND	ND	ND	ND	ND	ND	ND	ND	ND
16 (25)	2003	Watanabeet al.	+	ND	ND	ND	ND	+	ND	ND	+	+	ND	ND	ND	ND	ND	ND	ND	ND	ND
17 (3)	2003	Tochigiet al.	+	ND	ND	ND	ND	ND	ND	ND	ND	ND	ND	ND	ND	+	-	+	ND	ND	ND
18			+	ND	ND	ND	ND	ND	ND	ND	ND	-	ND	ND	ND	+	+	-	ND	ND	ND
19			+	ND	ND	ND	ND	ND	ND	ND	ND	+	ND	ND	ND	+	+	+	ND	ND	ND
20 (37)	2004	Tsunget al.	+	+	ND	ND	ND	ND	ND	ND	ND	ND	ND	+	ND	ND	-	-	ND	ND	+
21 (38)	2006	Yiğitet al.	+	ND	ND	ND	ND	ND	+	ND	ND	-	ND	-	ND	ND	ND	-	ND	ND	ND
22 (27)	2006	Kwon JEet al.	+	ND	ND	ND	ND	ND	ND	ND	ND	ND	ND	+	+	+	-	-	ND	+	ND
23 (39)	2007	Lazaroet al.	+	ND	ND	ND	ND	+	ND	ND	+	+	ND	ND	ND	ND	ND	ND	ND	ND	ND
24 (16)	2008	Tejerinaet al.	+	+	ND	ND	ND	+	ND	ND	ND	ND	+	+	ND	ND	ND	ND	ND	ND	ND
25 (23)	2008	Ozanet al.	+	ND	ND	ND	ND	+	ND	ND	+	-	+	+	ND	ND	ND	-	ND	-	-
26 (28)	2009	Zizi-Sermpetzoglouet al.	+	+	ND	ND	ND	+	ND	+	ND	ND	+	-	ND	-	-	-	-	ND	-
27 (31)	2009	Isonishi Set al.	+	ND	ND	ND	ND	ND	ND	ND	-	+	ND	ND	ND	ND	ND	ND	ND	ND	ND
28 (4)	2010	D’Antonioet al.	+	+	ND	ND	ND	+	ND	ND	ND	-	ND	-	ND	ND	ND	ND	ND	ND	ND
29[22	2011	Takahashiet al.	+	ND	ND	ND	ND	ND	ND	ND	ND	ND	ND	ND	ND	ND	ND	ND	ND	ND	ND
30 (40)	2011	Pandeyet al.	+	+	ND	ND	ND	ND	ND	ND	ND	ND	ND	ND	ND	ND	ND	ND	ND	ND	ND
31 (41)	2012	Liuet al.	+	-	ND	ND	ND	ND	ND	ND	-	ND	ND	ND	ND	+	ND	ND	ND	-	ND
32 (42)	2013	Wanget al.	+	+	ND	ND	ND	ND	ND	ND	ND	+	+	+	ND	ND	ND	ND	ND	+	+
33 (17)	2013	Sunget al.	-	-	ND	ND	ND	ND	ND	ND	ND	ND	ND	ND	ND	ND	ND	ND	ND	ND	ND
34 (18)	2013	Cascales Campos PAet al.	+	-	ND	ND	ND	ND	ND	ND	ND	+	ND	+	ND	ND	ND	ND	ND	ND	+
34 (43)	2015	Randolphet al.	+	+	ND	+	+	ND	ND	ND	ND	+	-	-	ND	ND	ND	-	+	+	ND
35 (29)	2015	Mazouzet al.	-	+	ND	ND	ND	ND	ND	ND	ND	ND	-	+	+	+	ND	-	ND	ND	ND
36 (44)	2016	Naffoujeet al.	+	+	+	ND	ND	ND	ND	ND	ND	ND	ND	+	+	+	ND	-	ND	ND	ND
37 (10)	2016	Lakhotiaet al.	+	ND	ND	ND	ND	ND	ND	ND	ND	ND	ND	ND	ND	ND	ND	ND	ND	ND	ND
38 (19)	2017	Mahmood H	+	+	+	-	+	ND	ND	ND	ND	ND	ND	ND	ND	ND	ND	ND	+	ND	ND
39 (24)	2017	Ishiguro Tet al.	-	-	ND	ND	ND	ND	ND	ND	ND	ND	+	+	ND	ND	ND	-	ND	ND	ND
40 (20)	2020	Choiet al.	+	+	ND	ND	ND	ND	ND	ND	ND	ND	ND	ND	ND	ND	ND	ND	ND	ND	ND
41 (45)	2020	Uribe Riveraet al.	+	+	+	+	ND	ND	ND	ND	ND	ND	ND	ND	ND	ND	ND	ND	ND	ND	ND
42 (21)	2020	Ghosh Jet al.	+	+	+	-	ND	ND	ND	ND	ND	ND	-	-	ND	ND	ND	-	ND	ND	-
43 (22)	2021	Liuet al.	-	+	ND	ND	ND	ND	ND	ND	ND	ND	ND	ND	ND	ND	ND	ND	ND	ND	ND
44			+	+	+	-	+	ND	ND	ND	ND	ND	ND	-	+	+	ND	ND	ND	ND	-
45 (26)	2023	Liet al.	+	+	ND	+	-	ND	ND	ND	ND	ND	+	-	ND	ND	ND	ND	+	ND	+
46 (15)	2024	Zhanget al.	+	-	+	+	ND	ND	ND	ND	ND	ND	+	ND	ND	ND	ND	ND	ND	ND	ND
47 (11)	2025	Zuo Yet al.	+	+	+	-	ND	ND	ND	ND	+	ND	ND	+	ND	ND	-	-	ND	-	+
48 (6)	2025	Zhang N.	+	+	+	+	ND	ND	ND	ND	ND	ND	+	-	ND	ND	ND	ND	ND	ND	+

+, Positive expression; -, Negative expression; ND, Not detected (undetected or unavailable data).

AFP, Alpha-fetoprotein; HepPar-1, Hepatocyte paraffin 1; Glypican-3, Glypican proteoglycan 3; SALL4, Spalt-like transcription factor 4; Arg1, Arginase 1; AAT, Alpha-1-antitrypsin; ACT, Alpha-1-antichymotrypsin; ALB, Albumin; CA125, Cancer antigen 125; CEA, Carcinoembryonic antigen; EMA, Epithelial membrane antigen; CK, Cytokeratin; AE1/AE3, Broad-spectrum epithelial cytokeratin; PLAP, Placental alkaline phosphatase; CD10, Membrane metallo-endopeptidase 10.

## 5 Diagnosis and differential diagnosis

Preoperative diagnosis of hepatoid carcinoma of the ovary (HCO) remains challenging, as it presents with non-specific clinical and imaging manifestations, even though most affected patients exhibit elevated serum alpha-fetoprotein (AFP). Histopathological examination combined with immunohistochemical profiling and molecular detection is still recognized as the gold standard for definitive diagnosis. HCO requires differential diagnosis from the following entities:

### 5.1 Ovarian yolk sac tumor

YST is a highly malignant germ cell tumor that predominantly affects young females, while HCO typically occurs in perimenopausal and postmenopausal women. Both tumors are associated with elevated serum AFP, but HCO is frequently accompanied by concurrent elevation of cancer antigen 125 (CA125). YST demonstrates diffuse positive staining for SALL4 and PLAP, which are absent in most cases of HCO. YST is typically negative for CK7, EMA, HepPar-1 and pCEA, whereas HCO commonly shows positive expression of these markers with diffuse membranous staining (28, 37, 40).

### 5.2 Metastatic hepatocellular carcinoma to the ovary

Differential diagnosis relies on medical history, serological testing, and radiological evaluation. Primary hepatocellular carcinoma (HCC) is typically associated with chronic liver disease and hepatic parenchymal lesions. Morphologically, hepatocellular carcinoma metastatic to the ovary (HCO) may be admixed with other adenocarcinoma components, a feature that is not observed in primary HCC. Immunohistochemically, AFP, HepPar1, Arg1, GPC3, CK8, and CK19 are universally positive in both tumors; CK7, CK19, and CK20 are expressed in a subset of HCO cases but are absent in primary HCC (41). Delayed ovarian recurrence of HCC occurring long after primary treatment further complicates the differential diagnosis between primary HCO and metastatic HCC to the ovary, requiring rigorous clinical screening (46).

### 5.3 Ovarian sex cord-stromal tumors

Leydig cell-rich ovarian sex cord-stromal tumors exhibit abundant eosinophilic cytoplasm, morphologically mimicking HCO, but are immunohistochemically AFP-negative and positive for WT1, α-inhibin, and calretinin (47). These tumors are often associated with endocrine symptoms due to hormonal hypersecretion. Cases of concurrent HCO and ovarian sex cord-stromal tumors have been reported, necessitating careful pathological discrimination (4).

### 5.4 Other epithelial ovarian carcinomas

The histological morphology and immunophenotype of other epithelial ovarian carcinomas are distinct from those of HCO, enabling reliable differentiation in most cases. Ovarian adenocarcinoma typically displays well-formed glandular structures, lacks expression of hepatocellular markers, is AFP-negative, and often shows positive expression of CEA, CA125, and CK7. Ovarian clear cell carcinoma is characterized by abundant clear cytoplasm and characteristic hobnail cells lining glandular/tubular structures, with consistent positivity for CK7, PAX8, and HNF1β and absence of hepatocellular markers. Rare cases of ovarian clear cell carcinoma may aberrantly express GPC3 and AFP (48, 49). Mixed tumors containing both HCO and other epithelial components present diagnostic challenges, requiring integrated evidence from morphological, immunohistochemical, and molecular analyses to achieve a definitive diagnosis (2, 3, 5).

## 6 Treatment

No standardized clinical guidelines or expert consensus are currently available for the management of hepatoid carcinoma of the ovary (HCO), and therapeutic strategies are mainly extrapolated from clinical experience with malignant epithelial ovarian tumors. Surgery is the cornerstone of primary treatment, with the extent of surgery determined by intraoperative exploration. All enrolled patients in included studies underwent surgical intervention, and over 80% received comprehensive surgical staging or cytoreductive surgery, including total hysterectomy, bilateral adnexectomy, omentectomy, and intraperitoneal tumor resection. Achieving R0 cytoreduction has been associated with a significant survival benefit for HCO patients.

Fertility-sparing comprehensive staging surgery has been reported in a 27-year-old patient of reproductive age, consisting of unilateral adnexectomy, pelvic and para-aortic lymphadenectomy, omentectomy, and peritoneal biopsy. No tumor recurrence was observed during the 2-year follow-up period (45), and this finding provides valuable evidence and clinical reference for fertility-sparing management for young patients with HCO. For gestational HCO, staged surgical intervention was performed: left oophorectomy and biopsy were conducted at 26 weeks of gestation, followed by cesarean section combined with hysterectomy, right oophorectomy, and omentectomy at 33 weeks of gestation. However, the patient died from extensive abdominal recurrence and metastasis 22 months after diagnosis (8).

Chemotherapy is the primary adjuvant therapeutic modality for HCO, including postoperative adjuvant chemotherapy, neoadjuvant chemotherapy, and salvage chemotherapy for recurrent disease. Approximately 85.41% (41/48) of reported HCO patients received systemic chemotherapy, and over 90% of these patients were treated with platinum-based combination regimens, primarily paclitaxel plus platinum. Nearly 70% of patients achieved partial or complete remission, with only one case terminating chemotherapy due to severe adverse reactions (22). Early case reports have described durable remissions in patients treated with combination chemotherapy consisting of cyclophosphamide or ifosfamide plus cisplatin (33, 35, 38). The BEP regimen (cisplatin + etoposide + bleomycin) has been administered to young patients or cases mixed with germ cell components, yielding both short-term and long-term remission (25, 31, 45). In contrast, regimens containing adriamycin or gemcitabine demonstrated limited efficacy, suggesting that HCO has inherent chemoresistance to these agents (20, 21, 23, 24).

Hyperthermic intraperitoneal chemotherapy (HIPEC), which has shown promising outcomes in advanced ovarian cancer with peritoneal dissemination (49, 50), achieved sustained tumor-free survival exceeding 22 months in one HCO patient treated with single-modality HIPEC postoperatively (44). Two patients who received HIPEC in combination with systemic paclitaxel-platinum combination chemotherapy achieved progression-free survival exceeding 25 months, supporting HIPEC as a promising therapeutic alternative for advanced HCO (11, 18).

Antiangiogenic agents inhibit tumor progression by suppressing pathological neovascularization. Bevacizumab has demonstrated robust clinical efficacy in first-line postoperative treatment, maintenance therapy, and recurrent disease management for patients with advanced epithelial ovarian cancer. Clinical evidence indicates that bevacizumab significantly extends progression-free survival and confers a modest but statistically significant improvement in overall survival in selected patient subgroups. Notably, a small case series reported in the literature described three patients with HCO who received bevacizumab in combination with standard chemotherapy; all achieved objective tumor responses, including partial or complete remission (11, 15, 24).

PARP inhibitors represent a novel targeted therapy for recurrent ovarian cancer. One HCO patient with a BLM heterozygous mutation received long-term maintenance therapy with niraparib plus anlotinib, achieving partial remission with 30-month progression-free survival (15). Sorafenib monotherapy failed to induce clinical remission in three HCO cases, suggesting the agent has limited efficacy for HCO (19, 21, 40). Further accumulation of clinical cases and molecular profiling data are required to identify actionable therapeutic targets and establish individualized precision treatment strategies for HCO.

Radiotherapy has limited routine application in clinical ovarian cancer management. Only 5 HCO cases have been reported to receive local or systemic radiotherapy: 4 patients with isolated metastatic lesions achieved effective local control via radiotherapy combined with chemotherapy (1, 20, 25); one early-stage patient underwent surgery plus radiotherapy without chemotherapy and maintained 3-year disease-free survival (28); one patient with mixed HCO and sex cord-stromal tumor obtained a favorable prognosis via postoperative chemoradiotherapy (4). Localized HCO is sensitive to radiotherapy, while pelvic-abdominal radiotherapy is rarely adopted as adjuvant therapy due to severe acute and chronic complications. Currently, local radiotherapy is reserved for palliative management of localized recurrent lesions.

## 7 Prognostic analysis

The overall prognosis of hepatoid carcinoma of the ovary (HCO) is extremely poor, as the disease exhibits a high propensity for early metastasis and a high recurrence rate. Over 63% of HCO patients are diagnosed at FIGO stage III–IV, with some studies reporting this proportion as high as 76.6%. More than 70% of patients with advanced-stage disease die within 2 years of diagnosis.

Given that the follow-up durations reported in existing literature range from 2 to 60 months, the observed mortality rate within the first two years may underestimate the true two-year mortality, because shorter follow-up intervals preclude accurate ascertainment of late occurring deaths. The median overall survival of patients with HCO is reported to be 2 years, with 1-year and 2-year survival rates of 83% and 53%, respectively (40, 43). Other studies report an overall survival range of 1 month to 5 years, with a 2-year survival rate of less than 50% and a 5-year survival rate of less than 10% (22). Stratified analysis demonstrates that median survival is 31 months for stage I–II disease, 10 months for stage III disease, and only 4 months for stage IV disease (15). Most HCO-related deaths are attributed to multiple organ failure, extensive systemic metastasis, or chemotherapy refractoriness.

Key prognostic determinants include clinical FIGO stage, the extent of cytoreductive surgery, and chemosensitivity or targeted drug responsiveness. Younger age is not associated with improved prognosis, indicating that the malignant biological behavior of HCO is independent of patient age. Tumor recurrence predominantly presents as abdominal dissemination, followed by hepatic, osseous, and pulmonary metastasis. Salvage treatment for recurrent HCO relies on multi-line chemotherapy, targeted therapy, and immunotherapy; while secondary surgery or radiotherapy may achieve short-term remission, durable clinical efficacy remains limited. Elevated serum alpha-fetoprotein (AFP) levels precede tumor recurrence detected by imaging modalities, making AFP a reliable dynamic biomarker for postoperative surveillance and therapeutic efficacy evaluation. However, clinicians should maintain vigilance for disease recurrence even in AFP-negative HCO cases.

## 8 Summary

Hepatoid carcinoma of the ovary (HCO) is a rare ovarian malignancy with distinct clinicopathological and molecular features. Definitive diagnosis depends on histopathological and immunohistochemical assessment combined with the clinical exclusion of metastatic lesions. Primary cytoreductive surgery followed by platinum-based chemotherapy remains the mainstay of current clinical management, although the overall prognosis for patients with HCO remains poor. Further clarification of the molecular pathogenesis of HCO and the development of targeted therapies are expected to improve long-term survival outcomes. Multicenter collaborative data accumulation of clinical cases and prospective cohort studies are urgently required to establish standardized diagnostic criteria and optimized clinical treatment pathways for HCO.
